# Tumor suppressor effect of an antibody on xenotransplanted sarcomatoid mesothelioma cells

**DOI:** 10.1111/1759-7714.14591

**Published:** 2022-08-02

**Authors:** Masayoshi Hasegawa, Yuki Hanamatsu, Chiemi Saigo, Yusuke Kito, Tamotsu Takeuchi

**Affiliations:** ^1^ Department of Pathology and Translational Research Gifu University Graduate School of Medicine Gifu Japan; ^2^ Department of Pathology Matsunami General Hospital Gifu Japan; ^3^ The United Graduate School of Drug Discovery and Medical Information Sciences, Gifu University Gifu Japan

**Keywords:** alternatively spliced isoform, AX10, mesothelioma, sarcolemma‐associated protein, therapeutic antibody

## Abstract

**Background:**

As mesothelioma generally has an unfavorable prognosis, further advances are needed to improve the outcomes in patients with mesothelioma. In the present study, we generated and characterized a monoclonal antibody that could inhibit mesothelioma cell proliferation in a xenotransplantation mouse model.

**Methods:**

We generated monoclonal antibodies by immunizing mice with cultured mesothelioma cells. These antibodies were then characterized by immunofluorescence staining, immunohistochemical staining, secondary antibody‐drug conjugate assay, antibody inoculation in a xenotransplantation mesothelioma mouse model, and mass spectrometry followed by small interfering RNA (siRNA) analysis. 5' rapid amplification of complementary DNA ends followed by sequencing was performed to deduce the amino acid sequences of the variable regions of the light and heavy chains of AX10.

**Results:**

An IgG2b κ‐type AX10 antibody against the cell surface membrane of sarcomatoid mesothelioma cells was generated. AX10 immunoreactivity was detected in 12 out of 22 different mesothelioma tissue specimens, but there was little AX10 immunoreactivity in a normal human tissue array. AX10 decreased Matrigel invasion by MPM‐1 cells but did not affect cell proliferation. Notably, AX10 significantly inhibited the proliferation of MPM‐1 cells xenotransplanted into Severe combined immunodeficiency‐Nonobese diabetic mice. Matrix‐assisted laser desorption ionization time‐of‐flight mass spectrometry followed by siRNA silencing indicated that AX10 reacted to a unique alternatively spliced isoform of sarcolemma‐associated protein. AX10 is composed of as yet unregistered amino acid sequences in its variable region.

**Conclusions:**

AX10 could have therapeutic potential for patients with sarcomatoid mesothelioma.

## Graphical abstract

Despite recent progress for diagnosis and therapies, the prognosis of mesothelioma, especially sarcomatoid mesothelioma, is unfavorable. In the present study, we developed monoclonal antibodies by immunizing cultured mesothelioma cells with focus on their potent therapeutic activity. Notably, AX10 significantly inhibited xenoplanted MPM‐1 cell growth in Severe combined immunodeficiency‐Nonobese diabetic mice. The present findings may indicate a potent therapeutic potential of AX10 antibody for sarcomatoid mesothelioma.

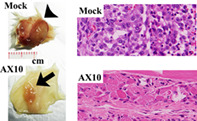

## INTRODUCTION

Mesothelioma is a mineral fiber‐related malignant tumor and is a worldwide healthcare problem.^1^ Although the peak incidence of mesothelioma is expected to occur before 2030 in high‐resource countries, the global incidence of mesothelioma continues to increase despite the ban on asbestos in many countries.^2^ Aside from asbestos, approximately 400 additional mineral fibers have not been banned, despite the fact that many of them are carcinogenic and related to mesothelioma.^3^, ^4^

Only a few treatment options are available for patients with mesothelioma. The benefit of surgery in mesothelioma remains controversial.^1^ Moreover, a significant proportion of patients with mesothelioma have unresectable tumors. Although radiotherapy has demonstrated a degree of benefit in symptom management, the role of radiotherapy beyond palliative care is debatable.^5^ In the past several decades, platinum/pemetrexed‐based chemotherapy has been the backbone for the treatment of patients with unresectable mesothelioma.^6^ Recently, combination immunotherapy using immune checkpoint inhibitors, i.e. ipilimumab and nivolumab, has appeared to provide a systemic therapy benefit for mesothelioma.^7^ However, even with this latest therapy, the median overall survival of patients with mesothelioma is only approximately 18 months.^7^ Therefore, further advances are needed to improve the prognosis of patients with mesothelioma.

In this study, we aimed to generate and characterize a monoclonal antibody that can inhibit the proliferation of mesothelioma cells *in vivo*. Our results indicated that a unique epitope of sarcolemma‐associated protein (also known as sarcolemmal membrane‐associated protein, SLMAP) could be a novel therapeutic target for patients with mesothelioma.

## MATERIALS AND METHODS

### Cells and cell culture

We used four mesothelioma cell lines. The ACC‐MESO‐1 mesothelioma cell line was obtained from the Riken Cell Bank (Tsukuba, Japan). The MPM‐1, ‐2, and ‐3 mesothelioma cell lines were established and maintained in our laboratory. Characterization of MPM‐1, ‐2, and ‐3 cells has been described previously.^8^ Cells were cultured in Dulbecco's modified Eagle's medium (DMEM) (Gibco Life Technologies) containing 10% heat‐inactivated fetal bovine serum (FBS) without any antibiotics. Cells were passaged for no more than 6 months after resuscitation.

### Generation of monoclonal antibodies

The experimental protocol was approved by the Animal Care Committee of Gifu University Graduate School of Medicine (Gifu, Japan; Approval no. 2021–149 and 2022‐087). A BALB/c mouse was immunized by weekly intraperitoneal injection of 1 × 10^6^ cultured cells from each of the MPM‐1, ‐2, and ‐3 mesothelioma cells. After the third immunization, splenocytes were fused with the myeloma cell line P3X63Ag8U1 (P3U1) according to the modified method of Koehler and Milstein.^9^, ^10^ Briefly, after fusion, cells were disseminated into two 24‐well plates in hypoxanthine‐aminopterin‐thymidine medium supplemented with 10% FBS. Ten days after fusion, the culture supernatant from each well was evaluated for the presence of cell surface membrane‐bound antibodies. Specifically, hybridoma clones with antibodies that reacted with intact MPM‐1, ‐2, and ‐3 cells were selected using immunofluorescence staining. Subsequently, three hybridoma clones with antibodies that reacted with mesothelioma tissues in a microarray were further selected. Finally, a hybridoma clone, which secreted antibodies that reacted minimally with normal microarrays, was selected. Antibody subclasses were determined using IsoQuick (EnviroLogix Inc.). The final antibody was purified from culture supernatants using Protein G‐Sepharose beads (GE Healthcare).

### 5′ rapid amplification of cDNA ends

We determined the nucleotide sequences encoding the variable regions of the light and heavy immunoglobulin chains of AX10 using the Takara rapid amplification of cDNA ends (RACE) complementary DNA (cDNA) Amplification Kit (Takara) according to the manufacturer's protocol. We employed the gene‐specific primers 5′‐ACTGAGGCACCTCCAGATGTTAACT‐3′ and 5′‐CTGGACAGGGATCCAGAGTTCCA‐3′ for amplification of the light and heavy chains of AX10, respectively.^11^

### Immunofluorescence staining

The cells were incubated with 1 μg/mL antibody at 4°C for 1 h. In the case of monoclonal antibody screening, the cells were incubated with culture supernatants of hybridomas at 4°C for 1 h. After washing with PBS, the cells were incubated with Alexa Fluor 488‐conjugated anti‐mouse antibody (1:200; Invitrogen) for 30 min at 4°C. After further washing with PBS, the cells were analyzed using a Guava easyCyte cell analyzer (Guava Technologies, Inc.).

### Immunohistochemical staining

Tissue microarrays composed of mesothelioma (Cat. No. MS801b) and Food and Drug Administration (FDA) normal organ tissue arrays (Cat. No. NBP2‐78057) were purchased from US Biomax and Novus Biologicals, respectively. A universal IHC/ISH control tissue array was purchased from Pantomics, Inc. All tissue samples were collected according to ethical standards and the Health Insurance Portability and Accountability Act (USA).

The tissues were immunostained with antibodies using an ImmPRESS polymerized reporter enzyme staining system (Vector Laboratories, Inc.) as previously reported.^12^

### Secondary antibody–drug conjugate assay in vitro

Anti‐murine IgG (Fc) secondary Ab conjugated to duocarmycin (Moradec) was used according to the manufacturer's protocol. Cytotoxicity was quantified with a fluorescein isothiocyanate (FITC)‐conjugated Annexin V and propidium iodide (PI) (PromoCell GmbH) assay as previously described.^13^

### Cell proliferation and Matrigel invasion assay

Cell proliferation was evaluated by counting the number of viable cells as previously described.^12^ Briefly, 1 × 10^4^ cells were cultured in standard 35 mm‐diameter tissue culture dishes (BD Biosciences) in triplicate. Live cells were counted after 24, 48, and 72 h.

Matrigel invasion activity was evaluated using 24‐well BD BioCoat Matrigel Invasion Chamber Plates (BD Biosciences) according to the manufacturer's protocol. The procedure has been described in detail previously.^13^ Briefly, 1 × 10^4^ cells were placed in the upper compartment of an invasion chamber. After incubation with DMEM containing 10% (lower chamber) or 2% (upper chamber) FBS, the cells on the lower surface of the filter were counted.

These assays were carried out in triplicate. Statistical analysis was performed by Student's *t*‐test for unpaired observation. Findings with *p*  < 0.05 were considered significant.

### Xenografts

The experimental protocol was approved by the Animal Care Committee of Gifu Graduate School of Gifu, Japan (Approval no. 2021–149). SCID‐NOD (NOD.CB17‐*Prkdc* ^*scid*^ /J) mice were purchased from Charles River Laboratories. MPM‐1 cells (3.3 × 10^6^) were injected subcutaneously into the soft tissue of the thighs of 10 Severe combined immunodeficiency‐Nonobese diabetic (SCID‐NOD) male mice (10 weeks old). Three days after the MPM‐1 cell xenotransplantation, the mice were randomly divided into two groups of five and then inoculated with or without 0.25 mg of AX10 antibody every week. The tumor dimensions were measured using calipers and the tumor volumes calculated based on the following equation: tumor volume (mm^3^) = 4/3π × [*a*/2] × [*b*/2]^2^, where *a* and *b* correspond to the longest and shortest measured diameters, respectively. The xenografts were excised, formalin‐fixed, paraffin‐embedded, and sectioned for histopathological analysis.

In addition, we examined the effect of AX10 compared to that of pemetrexed in the present xenoplant assay. For this experiment, 5.5 × 10^6^ MPM‐1 cells were subcutaneously injected into the soft tissue of the thigh of 15 10‐week‐old SCID‐NOD male mice. Three days after xenoplant, the mice were randomly divided into three groups of five mice, which were treated without agents, with AX10, or with pemetrexed. One group had 0.25 mg of AX10 antibody administered every week. The group treated with pemetrexed was given 0.5 mg intraperitoneally on days 7, 8, 9, 10, 11, 14, 15, 16, 17, and 18 according to the method described by Abu Lila et al.^14^ Pemetrexed was purchased from Merck.

### Peptide mass fingerprinting and MALDI‐TOF mass spectrometry

The procedure has been described before.^10^ Briefly, the purified AX10 antibody was bound to M‐270 epoxy magnetic beads (Thermo Fisher Scientific) according to the manufacturer's protocol. The AX10‐bound protein band was digested with trypsin (Promega) and subjected to matrix‐assisted laser desorption ionization time‐of‐flight (MALDI‐TOF) analysis (Microflex LRF 20; Bruker Daltonics), as described by Fernandez et al.^15^ Spectra were collected at 300 shots per spectrum over an *m*/*z* range of 700–4000 and calibrated through two‐point internal calibration using trypsin auto‐digestion peaks (*m*/*z* 842.5099, 2211.1046). The peak list was generated using Flex Analysis 3.0 software. The threshold used for peak‐picking was as follows: 500 for minimum resolution of monoisotopic mass and 6 for S/N. The search program MASCOT, developed by Matrixscience (http://www.matrixscience.com/), was used for protein identification via peptide mass fingerprinting. The following parameters were used for the database search: trypsin as the cleaving enzyme, a maximum of one missed cleavage, iodoacetamide (Cys) as complete modification, oxidation (Met) as partial modification, monoisotopic masses, and a mass tolerance of ±0.2 Da. The PMF acceptance criterion was probability scoring.

### siRNA‐mediated RNA interference and immunoblotting

The detailed procedure for siRNA silencing of target genes has been previously described.^13^ We employed small interfering RNAs (siRNAs) Cat No. AM16708, Assay ID 139737 and 139 738 (Thermo Fisher Scientific), to silence the sarcolemma‐associated protein gene, while a green fluorescent protein (GFP) siRNA duplex with the target sequence 5′‐CGGCAAGCUGACCCUGAAGUUCAU‐3′ was used as a nonsilencing control. The siRNAs were transfected into cells using lipofectamine RNAiMAX following the manufacturer's instructions (Invitrogen). The cells were used for subsequent studies at 72 h after transfection.

Immunoblotting was performed according to a previously described method,^10^ with the modification described by Towbin et al.^16^ Briefly, proteins were electrophoresed by sodium dodecyl sulfate‐polyacrylamide gel electrophoresis (SDS‐PAGE) and electroblotted onto polyvinylidene difluoride membranes (Immobilon‐P Transfer Membrane; Millipore). The membranes were then blocked with Block Ace (blocking milk; Yukijirushi).

The membranes were incubated with 0.5 μg/mL AX10 antibody and then reprobed by incubation with rabbit anti‐glyceraldehyde‐3‐phosphate dehydrogenase (GAPDH) antibody (Cat No. G9545; Sigma‐Aldrich). Chemiluminescent signals were detected using an Invitrogen iBright 1500 gel imaging system (Thermo Fisher Scientific).

In several of the experiments, we also used commercially available conventional rabbit antibody to sarcolemma‐associated protein (Cat No. 25220‐1‐AP; Proteintech Group Inc.).

## RESULTS

### Generation of AX10 antibody

**FIGURE 1:**
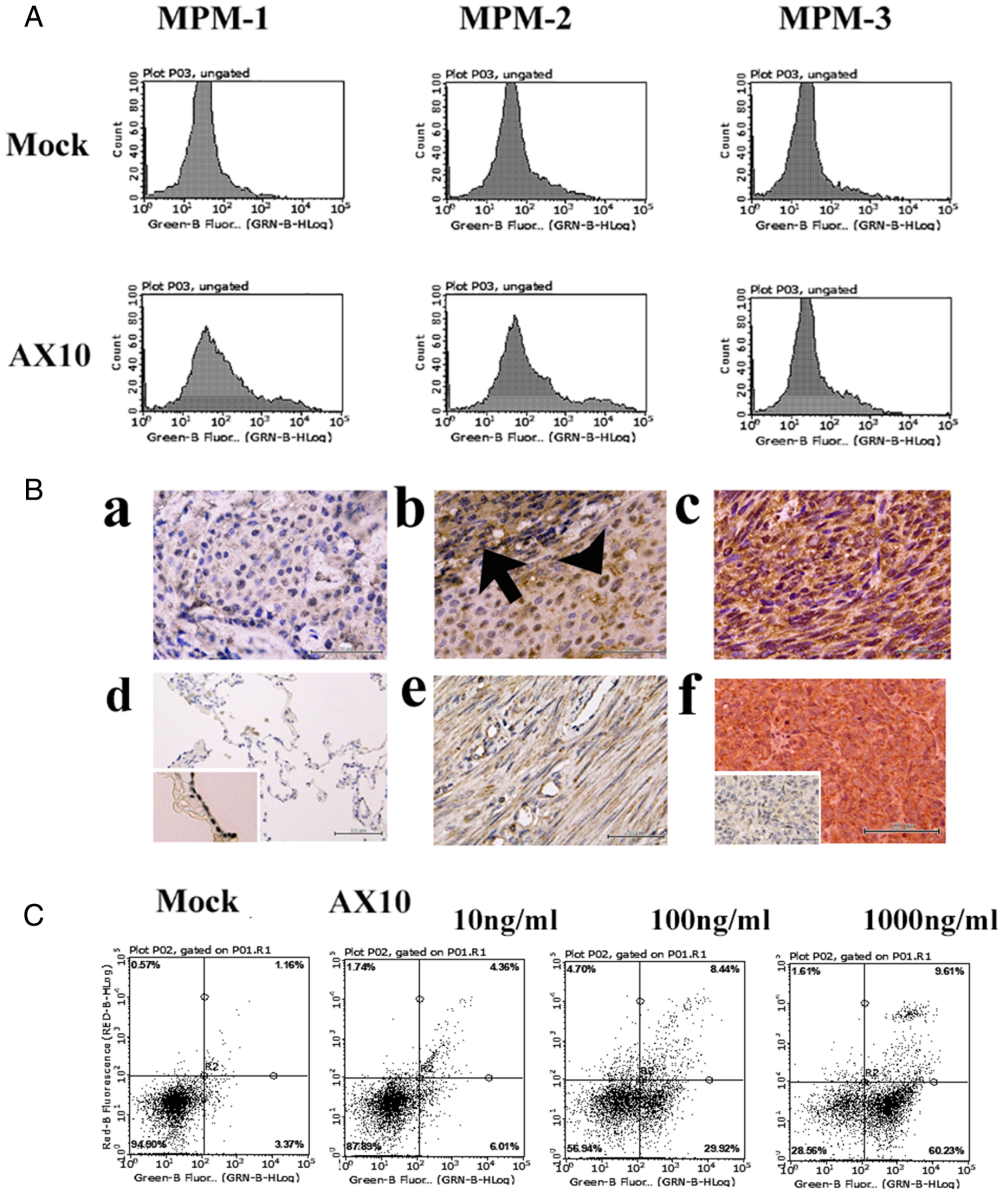
Representative immunofluorescent staining of cultured mesothelioma cells using AX10 antibody (a), immunohistochemical staining (b), and secondary antibody‐drug conjugate assay in vitro (c). (a) AX10 immunoreactivity in MPM‐1, −2, and −3 cells, representing sarcomatoid, epithelioid, and biphasic type mesothelioma, respectively. All MPM‐1, −2, and −3 cells exhibited AX10 antibody immunoreactivity at the cell surface. The staining was analyzed using a Guava easyCyte cell analyzer and accompanying software to obtain a one‐parameter log histogram. (b) AX10 immunoreactivity in various mesothelioma tissue specimens. Weak or no AX10 immunoreactivity was detected in five out of 10 epithelioid mesothelioma tissues (a). One out of five biphasic mesotheliomas exhibited AX10 immunoreactivity in spindle sarcomatoid components (arrow) but weak immunoreactivity in epithelioid components (arrowhead) (b). Five out of six sarcomatoid mesothelioma tissues exhibited strong AX10 immunoreactivity (c). Little AX10 immunoreactivity was detected in normal human tissues. No significant AX10 immunoreactivity was detected in the lung (d) (pleural mesothelial cells; insert) tissue specimens. Weak AX10 immunoreactivity was detected in myofibrous cells in the uterus (e). We did not detect any significant AX10 immunoreactivity in the brain, liver, or kidney, whereas strong AX10 immunoreactivity was observed in a nonmelanocytic (hypomelanocytic) melanoma tissue sample that was supplementally included in the microarray (f) (staining without AX10 antibody; insert). (c) MPM‐1 sarcomatoid mesothelioma cells were incubated with AX10 at 10, 100, and 1000 ng/mL followed by incubation with anti‐murine IgG (Fc) antibody conjugated to duocarmycin. Representative staining with Annexin V‐PI is presented. Note the dose‐dependent Annexin V‐positive and PI‐negative apoptotic MPM‐1 cells in the presence of AX10 antibody

A monoclonal antibody, designated AX10, reacted with the cell surface membrane of MPM‐1, ‐2, ‐3, and ACC‐MESO‐1 cells (Figure 1a and Supporting Information S1). Preliminary immunohistochemical staining using hybridoma cell culture supernatants demonstrated that AX10 reacted with a microarray composed of mesothelioma tissues, but there was little AX10 reactivity with normal human tissues. Therefore, we further characterized AX10 after limiting dilution.

AX10 was determined to be of the IgG2b κ type, and it was purified using a Protein G column before being further characterized. Representative data of immunohistochemical staining using purified AX10 are shown in Figure 1b. Notably, AX10 immunoreactivity was detected in 12 out of 22 different mesothelioma tissue specimens. Interestingly, AX10 preferentially reacted with sarcomatoid mesothelioma cells rather than epithelioid cells (Figure 1b(a–c)). AX10 immunoreactivity was detected in five out of six sarcomatoid mesothelioma tissue specimens, whereas it was detected in five out of 10 epithelioid and one out of five biphasic mesothelioma tissue specimens. By contrast, little AX10 immunoreactivity was detected in an FDA normal organ tissue array. No significant AX10 immunoreactivity was detected in the lung (Figure 1b(d)), pleural mesothelial cells (Figure 1b(d), insert), liver, kidney, or brain. Weak AX10 immunoreactivity was detected in myofibrocytic cells in the uterus (Figure 1b(e)). Notably, strong AX10 immunoreactivity was observed in amelanotic melanoma (hypomelanotic melanoma), which was supplementally included in the microarray (Figure 1b(f)).

Moreover, AX10 appeared to induce apoptosis in sarcomatoid MPM‐1 cells by secondary antibody‐drug conjugate assay (Figure 1c). These results indicate that AX10 was successfully internalized into MPM‐1 cells after binding to the cell surface membrane.

The cDNA sequences of the variable regions of the light and heavy chains of AX10 are shown in Supporting Information Table S1 (accession numbers LC715420 and LC715419, respectively). Notably, the deduced amino acid sequences of the variable regions of the light and heavy chains of AX10 were unregistered in the public database.

### AX10 inhibited Matrigel invasion activity of MPM‐1 cells but did not affect cell proliferation

**FIGURE 2:**
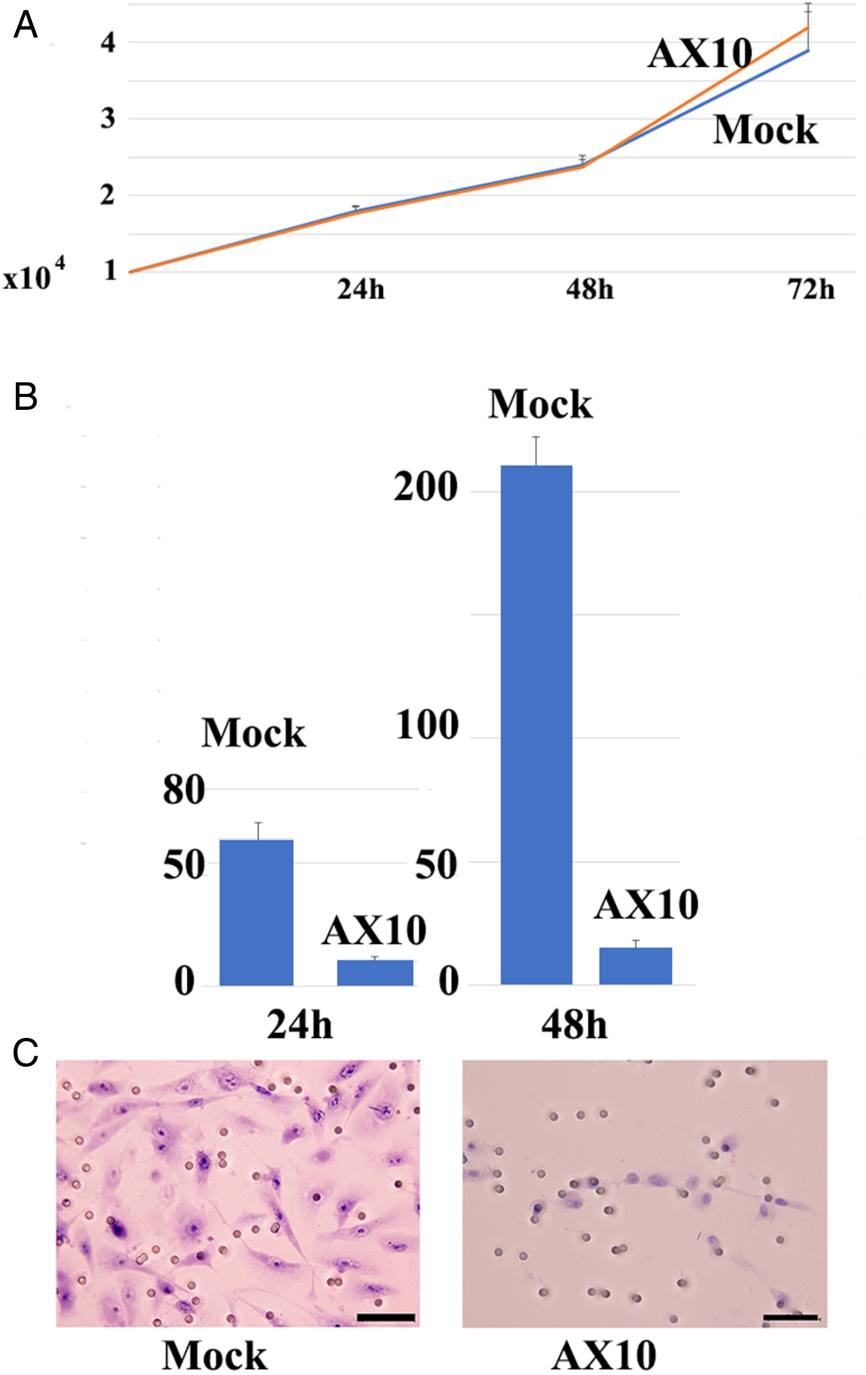
AX10 does not affect cell proliferation, but significantly decreases Matrigel invasion activity of MPM‐1 sarcomatoid mesothelioma cells in vitro. (a) Representative cell proliferation assay. At 24 h, the cell number was 1.80 ± 0.10 (mock) and 1.77 ± 0.06 (AX10). Respective numbers at 48 h were 2.40 ± 0.10 (mock) and 2.37 ± 0.12 (AX10), while at 72 h they were 3.90 ± 0.20 (mock) and 4.20 ± 0.61 (AX10). The data represent means ± SD from triplicate assays (Student's *t*‐test, *p* > 0.5). (b) AX10 significantly reduced Matrigel invasion activity of MPM‐1 cells (Student's *t*‐test, *p* < 0.01). The number of invading cells was 59.7 ± 7.02 (mock) and 10.3 ± 1.52 (AX10) at 24 h, and 210.7 ± 11.4 (mock) and 15.0 ± 3.00 (AX10) at 48 h. Data from triplicate assays are expressed as means ± SD (*n* = 3). (c) Cells that migrated to the lower surface of the membrane are shown (48 h). Original magnification, ×100

Next, we investigated whether AX10 altered the pathobiological property of MPM‐1 cells in vitro. Although AX10 had no significant effect on MPM‐1 cell proliferation (Figure 2a), Matrigel invasion activity was significantly reduced by AX10 (Figure 2b,c).

### AX10 suppressed MPM‐1 tumor growth in xenotransplanted mice

**FIGURE 3:**
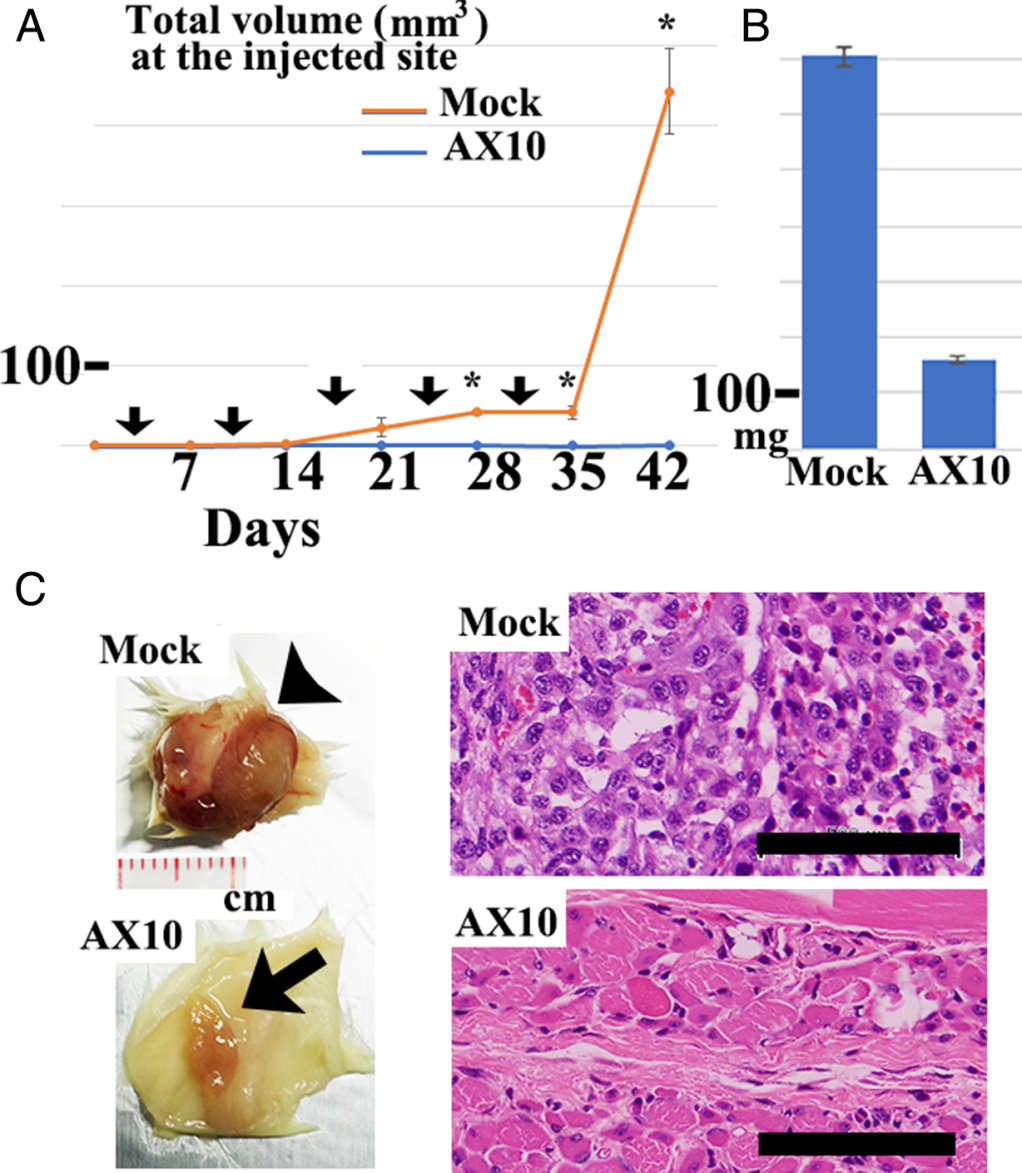
Inhibitory effect of AX10 on MPM‐1 xenotransplanted sarcomatoid mesothelioma cell proliferation. (a) Inoculation of AX10 antibody delayed the growth of xenotransplanted MPM‐1 sarcomatoid mesothelioma tumors. On day 0, SCID‐NOD mice were subcutaneously implanted with MPM‐1 cells. The following day, day 3, the mice were administered AX10 antibody or vehicle only by intraperitoneal injection and weekly thereafter as indicated by arrows. Values are represented as means ± standard error for *n* = 5 mice. Statistical significance was measured by a two‐sided unpaired Student's *t*‐test (*p < 0.01). (b) On day 42, the xenotransplanted tumors were excised to determine their weight. Total tumor weights are represented as means ± standard error for *n* = 5 mice. Statistical significance was measured by a two‐sided unpaired Student's *t*‐test (*p* < 0.01). (c) Gross and histological appearance of a representative xenotransplanted tumor. Arrowhead indicates the tumor without AX10 antibody, while the arrow indicates the small tumor remaining following weekly AX10 injection. Note the elimination of tumor cells, which were histologically replaced by regenerative muscle in mice inoculated with AX10 antibody. Scale bar indicates 100 μm

We also examined the effect of AX10 on xenotransplanted MPM‐1 cell proliferation. The results are summarized in Figure 3. Notably, the proliferation of xenotransplanted MPM‐1 cells was impaired by inoculation of AX10 antibody with statistical significance (*p* < 0.01). In the present study, pemetrexed did not significantly suppress MPM‐1 tumor growth (Supporting Information Figure S2).

### AX10 bound to an epitope on sarcolemma‐associated protein

**FIGURE 4:**
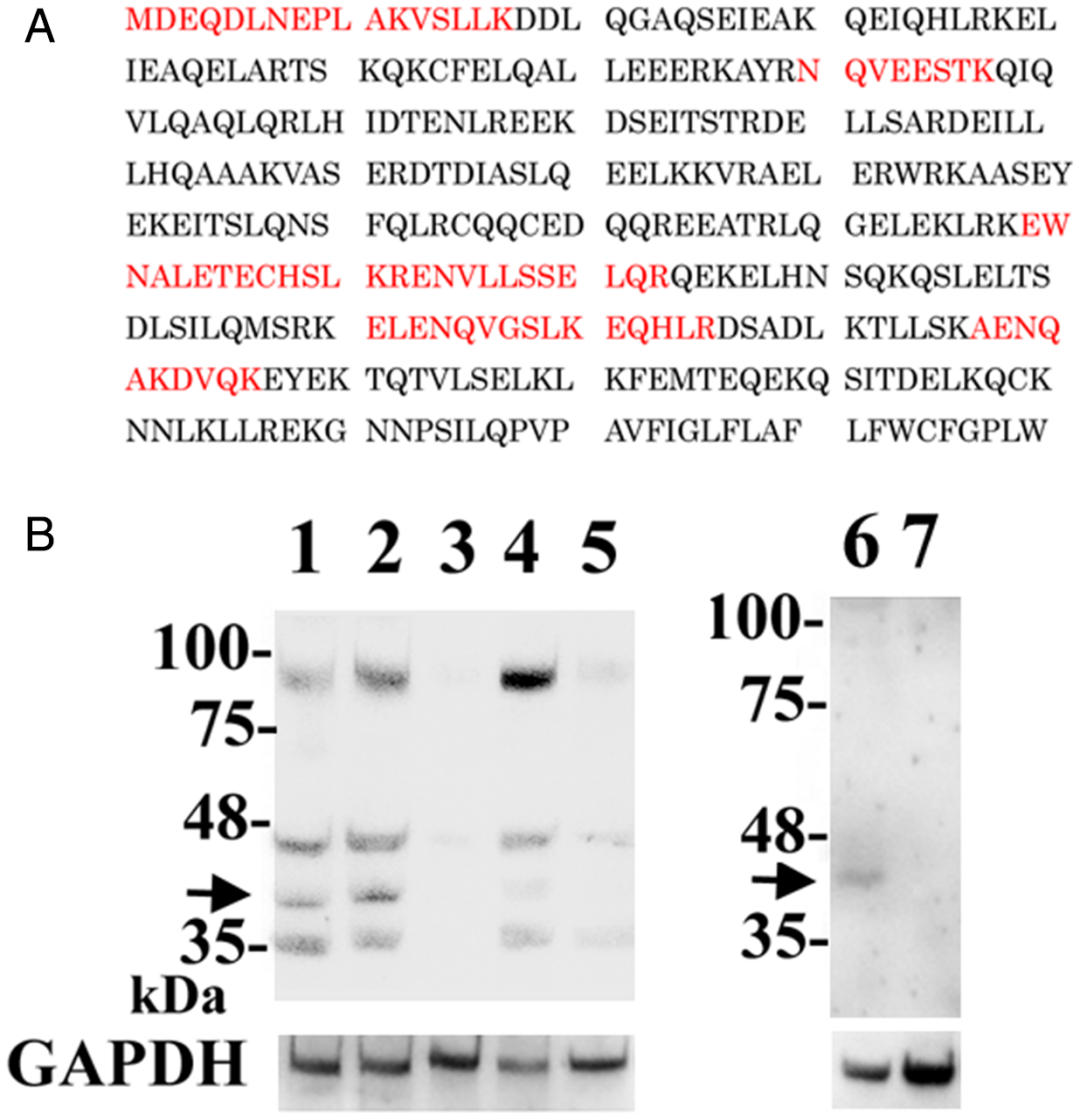
Characterization of the AX10 antigen. (a) Peptide coverage is present in human SLMAP. Matching peptide is indicated in red font. (b) Left (lanes 1 to 5), immunoblotting using a commercially available anti‐SLMAP antibody. Protein bands of approximately 90, 45, 40, and 35 kDa were detected in lysates of MPM‐1 cells (lane 1). The same four bands were observed in lysates of MPM‐1 cells treated with GFP‐siRNA (lane2), but not in lysates of MPM1 cells treated with SLMAP‐siRNA (lane 3). Note the small amount of 40 kDa band in SW480 colon cancer cells (lane 4). SLMAP‐siRNA treatment also eliminated the 90, 45, and 35 kDa SLMAP protein bands in SW480 cells (lane 5). Right (lane 6 and 7), immunoblotting using AX10 antibody. An approximately 40 kDa band was observed in lysates of MPM‐1 cells treated with GFP‐siRNA (lane 6), but not in lysates of MPM‐1 cells treated with SLMAP‐siRNA (lane 7). Protein loading was evaluated by anti‐GAPDH immunoblotting

We characterized the AX10 antigen. AX10 antigen was purified from MPM‐1 cells using AX10‐binding M‐270 epoxy magnetic beads. The extracted protein band with an apparent molecular weight of 40 kDa was isolated and analyzed by peptide mass fingerprinting by MALDI‐TOF assay. The identified trypsin‐digested peptides appeared to be components of SLMAP (Figure 4a).

We then knocked down SLMAP expression at the protein level using siRNA‐mediated silencing in MPM‐1 cells. Notably, siRNA targeting and immunoblotting confirmed that AX10 reacted with SLMAP in MPM‐1 cells.

Subsequent immunohistochemical staining demonstrated that immunoreactivity using a commercially available antibody against SLMAP was found in various normal tissue arrays, including lung and muscle tissue, which were not stained by AX10 (Supporting Information Figure S3). Notably, AX10‐positive sarcomatoid mesothelioma tissues also stained with a commercially available antibody against SLMAP, whereas AX10‐negative epithelioid mesothelioma tissues did not stain with a commercially available antibody against SLMAP. These results indicate that AX10 reacted with a unique epitope of SLMAP, which was expressed in mesothelioma cells, particularly sarcomatoid mesothelioma cells.

## DISCUSSION

In the present experiment, we generated a monoclonal antibody, designated AX10, that reacted with many mesothelioma tissues, especially the sarcomatoid type. By contrast, little AX10 immunoreactivity was detected in the normal tissues that were examined (Figure 1b). Notably, AX10 suppressed Matrigel invasion activity and the proliferation of xenotransplanted sarcomatoid mesothelioma cells (Figures 2 and 3). Moreover, AX10 antibody was internalized and induced apoptosis in MPM‐1 mesothelioma cells by secondary antibody‐drug conjugate assay (Figure 1c). Therefore, modification of AX10, for example by conjugation to a drug, has the potential to enhance the tumor suppressor function of AX10.

The present findings indicate that AX10 reacts with SLMAP protein. SLMAPs comprise a membrane protein family of alpha‐helical coiled‐coil proteins with a transmembrane domain encoded by a single gene mapped to human chromosome 3p14.3–21.2.^17^, ^18^ The *SLMAP* gene undergoes multiple alternative splicing events to generate many isoforms with molecular weights of 35, 45, 63–66, and 83–91 kDa. In addition, several splice variants of SLMAPs exhibit developmental and tissue‐specific expression.^19^ SLMAP is known to be overexpressed in sarcoma, although it is not known which splicing form of SLMAP is overexpressed.^20^, ^21^ Notably, strong AX10 immunoreactivity was detected in melanoma cells in tissue microarrays (Figure 1b(f)). We hypothesize that AX10 may recognize a unique epitope on an alternatively spliced form of SLMAP that is preferentially expressed in sarcomatoid mesothelioma cells and several other malignant tumors. Since the functional diversity of SLMAP is thought to be dependent on alternative splicing, the SLMAP isoform recognized by AX10 may contribute to tumorigenesis, including that of mesothelioma.

Although SLMAP was initially identified as a component of the cardiac sarcolemma involved in myoblast fusion and muscle contraction, it is now widely accepted that SLMAP and its splicing forms have a pleiotropic biological function. Notably, SLMAP is also a subunit of the STRIPAK PP2A phosphatase complex, which participates in the Hippo signaling axis.^22^ Loss of function of NF2, which is one of the regulators of the Hippo pathway, is observed in 19–50% of malignant mesotheliomas.^23^

Further extensive studies are needed to unravel the pathobiological role of SLMAP in mesothelial carcinogenesis.

In conclusion, the present findings indicate that AX10 is an antibody with therapeutic potential for patients with sarcomatoid mesothelioma.

## AUTHOR CONTRIBUTIONS

Tamotsu Takeuchi participated in the design of the study, data interpretation, and manuscript drafting. Masayoshi Hasegawa, Yuki Hanamatsu, Chiemi Saigo, and Yusuke Kito performed the experiments. All authors read and approved the manuscript and agreed to their individual contributions prior to submission.

## CONFLICT OF INTEREST

We have no conflicts of interest to declare.

## ETHICS APPROVAL

Informed consent was obtained from all the study participants or their authorized representatives. The study was conducted in accordance with the ethical standards set out in the 1975 Helsinki Declaration.

## Supporting information


**Supporting Information Table S1** Nucleotide sequences of the variable regions of the light and heavy chains of AX10. Bold “ATG” indicates the start codon, the underlined sequences anneal to the specific antisense primer for 5′‐RACE.

**Supporting Information Figure S1** AX10 immunoreactivity was found in MPM‐1, −2, −3, and ACC‐MESO‐1 cells. The staining was analyzed using a Guava easyCyte cell analyzer and accompanying software to obtain a one‐parameter log histogram.

**Supporting Information Figure S2** Tumor suppression function of AX10 or pemetrexed in the present xenoplant assay. A Tukey's *t*‐test confirmed there was a statistically significant difference, *p* < 0.05, between AX10 and the pemetrexed or mock groups on day 35. There was no significant difference between the pemetrexed and mock groups (*p* > 0.05, Tukey's *t*‐test).

**Supporting Information Figure S3** Comparison of the immunoreactivity of a commercially available antibody against SLMAP (a, c, e, and g) and AX10 antibody (b, d, f, and h). (a) and (b) Immunoreactivity was found in muscle cells (indicated with *) and infiltrated lymphocytes (indicated with an arrow) using a commercially available anti‐SLMAP antibody (Proteintech, Cat No. 25220‐1‐AP), whereas AX10 immunoreactivity was not found in these cells. (c) and (d) Nontumorous lung epithelial cells exhibited immunoreactivity with a commercially available anti‐SLMAP antibody (indicated with an arrow), but not with AX10. (e) and (f) Sarcomatoid mesothelioma cells were stained by both a commercially available antibody against SLMAP and AX10. Note the strong immunoreactivity with AX10 compared to that with the commercially available anti‐SLMAP antibody. (g) and (h) Weak immunoreactivity using both a commercially available anti‐SLMAP antibody and AX10 in epithelioid mesothelioma. Note the immunoreactivity in vascular wall cells (indicted with an arrow) in (g), but not in (h).
