# Supplementary material for: Tumor suppressor effect of an antibody on xenotransplanted sarcomatoid mesothelioma cells
Source: Thorac Cancer. 2022 Aug 2;13(18):2566–73. doi: 10.1111/1759-7714.14591 (PMC9475231; doi:10.1111/1759-7714.14591)
Supplement: Supplementary file 1 — Supporting Information Table S1 Nucleotide sequences of the variable regions of the light and heavy chains of AX10. Bold “ATG” indicates the start codon, the underlined sequences anneal to the specific antisense primer for 5′‐RACE. [file TCA-13-2566-s001.docx]

**AX10 light chain variable region ATG**AGGTTCTCTGCTCAGCTTCTGGGGCTGCTTGTGCTCTGGATCCCTGGATCCACTGCAGATATTGTGATGACGCAGGCTGCCTTCTCCAATCCAGTCACTCTTGGAACATCAGCTTCCATCTCCTGCAGGTCTAGTAAGAATCTCCTACATAGTAATGGCATCACTTATTTGTATTGGTATCTGCAGAGGCCAGGCCAGTCTCCTCAGCTCCTGATATATCGGGTGTCCAATCTGGCCTCAGGAGTCCCAAACAGGTTCAGTGGCAGTGAGTCAGGAACTGATTTCACACTGAGAATCAGCAGAGTGGAGGCTGAGGATGTGGGTGTTTATTACTGTGCTCAACTGCTAGAACTCCCGTACACGTTCGGAGGGGGGACCAAGCTGGAAATAAAACGGGCTGATGCTGCACCAACTGTATCCATCTTCCCACCATCCAGTGAGCAGTTAACATCTGGAGGTGCCTCAGT

**Deduced amino acid sequence of variable region of AX10 light chain**

MRFSAQLLGLLVLWIPGSTADIVMTQAAFSNPVTLGTSASISCRSSKNLL

HSNGITYLYWYLQRPGQSPQLLIYRVSNLASGVPNRFSGSESGTDFTLRI

SRVEAEDVGVYYCAQLLELPYTFGGGTKLEIKRADAAPTVSIFPPSSEQL

TSGGAS

**AX10 heavy chain variable region**

**ATG**GAATGGAGCTGGGTCTTTCTCTTCCTCCTGTCAGTAACTGCAGGTGTCCAATCCCAGGTTCAACTGCAGCAGTCTGGGGCTGAGCTGGTGAGGCCTGGGGCTTCAGTGAAGCTGTCCTGCAAGGCTTTGGGCTACACATTTACTGACTATGAAATGCACTGGGTGAAGCAGACACCTGTGCATGGCCTGGAATGGATTGGAGCTATTCATCCAGGAAGTGGTGGTACTGCCTACAATCAGAAGTTCAAGGGCAAGGCCACACTGACTGCAGACAAATCCTCCAGCACAGCCTACATGGAGCTCAGCAGCCTGACATCTGAGGACTCTGCTGTCTATTACTGTACACCCTACCCGTTTGCTTACTGGGGCCAAGGGACTCTGGTCACTGTCTCTGCAGCCAAAACGACACCCCCATCTGTCTATCCACTGGCCCCTGGATCTGCTGCCCAAACTAACTCCATGGTGACCCTGGGATGCCTGGTCAAGGGCTATTTCCCTGAGCCAGTGACAGTGACCTGGAACTCTGGATCCCTGTCCAG

**Deduced amino acid sequence of variable region of AX10 heavy chain**

MEWSWVFLFLLSVTAGVQSQVQLQQSGAELVRPGASVKLSCKALGYTFTD

YEMHWVKQTPVHGLEWIGAIHPGSGGTAYNQKFKGKATLTADKSSSTAYM

ELSSLTSEDSAVYYCTPYPFAYWGQGTLVTVSAAKTTPPSVYPLAPGSAA

QTNSMVTLGCLVKGYFPEPVTVTWNSGSLS
