# Supplementary figures and images for: Tumor suppressor effect of an antibody on xenotransplanted sarcomatoid mesothelioma cells
Source: Thorac Cancer. 2022 Aug 2;13(18):2566–73. doi: 10.1111/1759-7714.14591 (PMC9475231; doi:10.1111/1759-7714.14591)

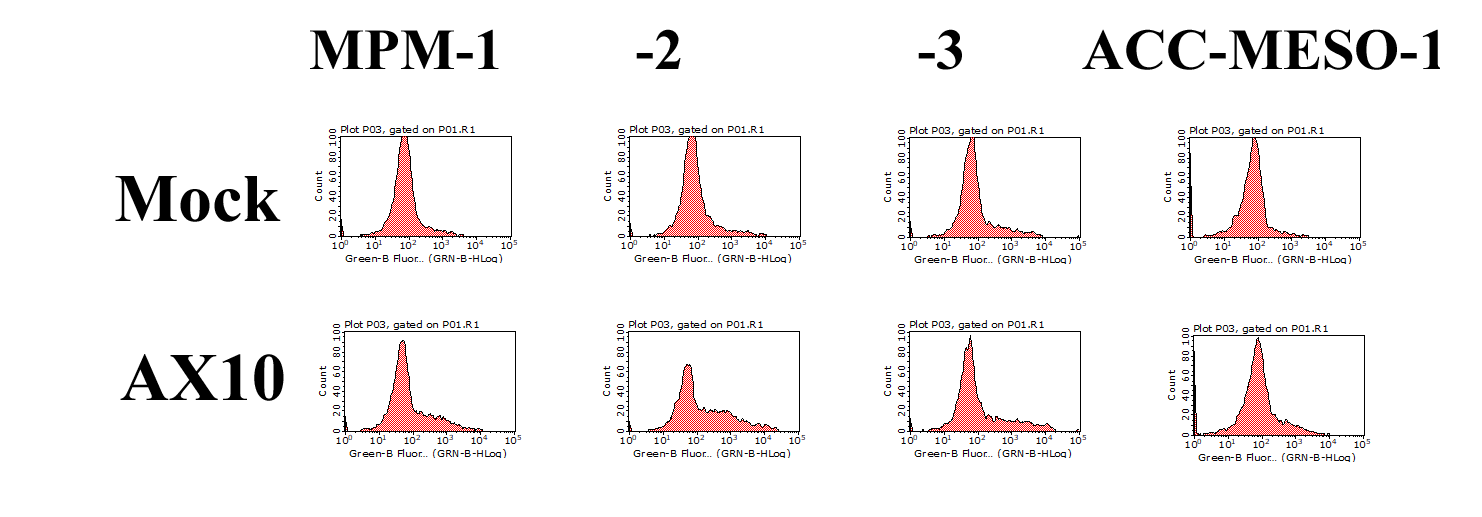

Supplement: Supplementary file 2 — Supporting Information Figure S1 AX10 immunoreactivity was found in MPM‐1, −2, −3, and ACC‐MESO‐1 cells. The staining was analyzed using a Guava easyCyte cell analyzer and accompanying software to obtain a one‐parameter log histogram. [file TCA-13-2566-s002.tif]

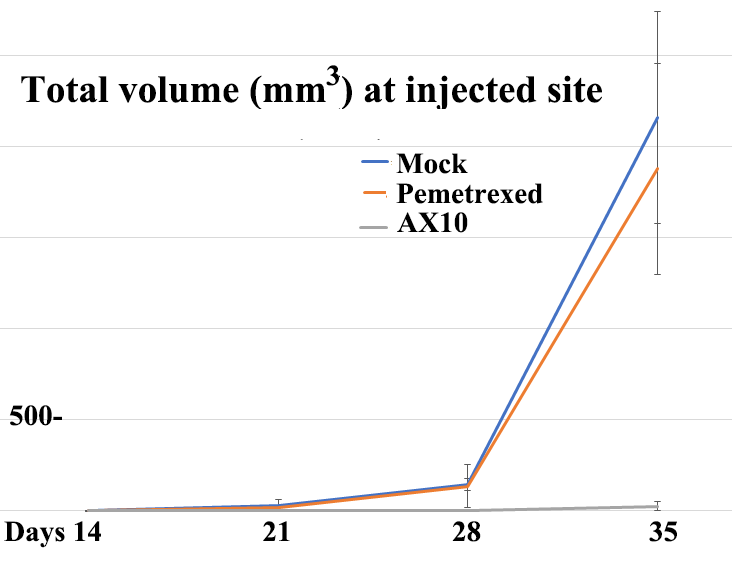

Supplement: Supplementary file 3 — Supporting Information Figure S2 Tumor suppression function of AX10 or pemetrexed in the present xenoplant assay. A Tukey's t‐test confirmed there was a statistically significant difference, p < 0.05, between AX10 and the pemetrexed or mock groups on day 35. There was no significant difference between the pemetrexed and mock groups (p > 0.05, Tukey's t‐test). [file TCA-13-2566-s003.tif]

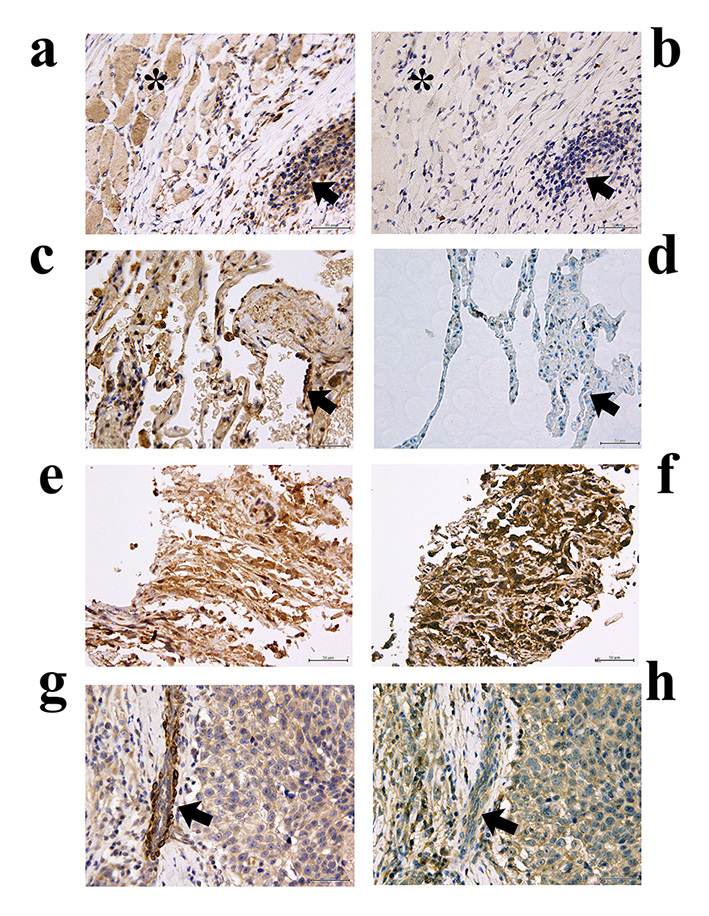

Supplement: Supplementary file 4 — Supporting Information Figure S3 Comparison of the immunoreactivity of a commercially available antibody against SLMAP (a, c, e, and g) and AX10 antibody (b, d, f, and h). (a) and (b) Immunoreactivity was found in muscle cells (indicated with *) and infiltrated lymphocytes (indicated with an arrow) using a commercially available anti‐SLMAP antibody (Proteintech, Cat No. 25220‐1‐AP), whereas AX10 immunoreactivity was not found in these cells. (c) and (d) Nontumorous lung epithelial cells exhibited immunoreactivity with a commercially available anti‐SLMAP antibody (indicated with an arrow), but not with AX10. (e) and (f) Sarcomatoid mesothelioma cells were stained by both a commercially available antibody against SLMAP and AX10. Note the strong immunoreactivity with AX10 compared to that with the commercially available anti‐SLMAP antibody. (g) and (h) Weak immunoreactivity using both a commercially available anti‐SLMAP antibody and AX10 in epithelioid mesothelioma. Note the immunoreactivity in vascular wall cells (indicted with an arrow) in (g), but not in (h). [file TCA-13-2566-s004.jpg]
